# High summer temperatures amplify functional differences between coral‐ and algae‐dominated reef communities

**DOI:** 10.1002/ecy.3226

**Published:** 2020-12-27

**Authors:** Florian Roth, Nils RAdecker, Susana Carvalho, Carlos M. Duarte, Vincent Saderne, Andrea Anton, Luis Silva, Maria Ll. Calleja, XosÉ Anxelu G. MorÁn, Christian R. Voolstra, Benjamin Kürten, Burton H. Jones, Christian Wild

**Affiliations:** ^1^ Red Sea Research Center King Abdullah University of Science and Technology (KAUST) Thuwal 23955 Saudi Arabia; ^2^ Baltic Sea Centre Stockholm University Stockholm 10691 Sweden; ^3^ Faculty of Biological and Environmental Sciences Tvärminne Zoological Station University of Helsinki Helsinki 00014 Finland; ^4^ Department of Biology University of Konstanz Konstanz 78457 Germany; ^5^ Laboratory for Biological Geochemistry School of Architecture Civil and Environmental Engineering École Polytechnique Fédérale de Lausanne (EPFL) Lausanne 1015 Switzerland; ^6^ Computational Biology Research Center King Abdullah University of Science and Technology (KAUST) Thuwal 23955 Saudi Arabia; ^7^ Department of Climate Geochemistry Max Planck Institute for Chemistry (MPIC) Mainz 55128 Germany; ^8^ Project Management Jülich Jülich Research Centre GmbH Rostock 52425 Germany; ^9^ Marine Ecology Faculty of Biology and Chemistry University of Bremen Bremen 28359 Germany

**Keywords:** activation energy, biogeochemical cycling, climate change, community budget, ecosystem functioning, regime shifts

## Abstract

Shifts from coral to algal dominance are expected to increase in tropical coral reefs as a result of anthropogenic disturbances. The consequences for key ecosystem functions such as primary productivity, calcification, and nutrient recycling are poorly understood, particularly under changing environmental conditions. We used a novel in situ incubation approach to compare functions of coral‐ and algae‐dominated communities in the central Red Sea bimonthly over an entire year. In situ gross and net community primary productivity, calcification, dissolved organic carbon fluxes, dissolved inorganic nitrogen fluxes, and their respective activation energies were quantified to describe the effects of seasonal changes. Overall, coral‐dominated communities exhibited 30% lower net productivity and 10 times higher calcification than algae‐dominated communities. Estimated activation energies indicated a higher thermal sensitivity of coral‐dominated communities. In these communities, net productivity and calcification were negatively correlated with temperature (>40% and >65% reduction, respectively, with +5°C increase from winter to summer), whereas carbon losses via respiration and dissolved organic carbon release more than doubled at higher temperatures. In contrast, algae‐dominated communities doubled net productivity in summer, while calcification and dissolved organic carbon fluxes were unaffected. These results suggest pronounced changes in community functioning associated with coral‐algal phase shifts. Algae‐dominated communities may outcompete coral‐dominated communities because of their higher productivity and carbon retention to support fast biomass accumulation while compromising the formation of important reef framework structures. Higher temperatures likely amplify these functional differences, indicating a high vulnerability of ecosystem functions of coral‐dominated communities to temperatures even below coral bleaching thresholds. Our results suggest that ocean warming may not only cause but also amplify coral–algal phase shifts in coral reefs.

## Introduction

Community shifts and the ongoing loss of biodiversity (Brondizio et al. 2019) are altering the productivity and biogeochemistry of many ecosystems globally (Middleton and Grace 2004, Hooper et al. 2012, Naeem et al. 2012). These changes compound with local and global environmental perturbations, which can accelerate the alteration of essential ecosystem processes (Balvanera et al. 2006, Stachowicz et al. 2007). Thermal stress caused by climate change is, thereby, likely to exhibit the most substantial impact (Stillman 2019).

Tropical coral reefs are hotspots of biodiversity that provide various ecosystem services that are supported by one or more metabolic or biogeochemical functions (e.g., primary production, calcification, organic matter fluxes, and nutrient cycling; Moberg and Folke 1999). Many of these processes are primarily driven by scleractinian corals, the “ecosystem engineers” of tropical reefs (Wild et al. 2011). However, the combination of global and local anthropogenic stressors has caused extensive coral mortality and subsequent shifts from complex coral‐dominated communities to simplified communities with a predominance of filamentous turf‐ and macroalgae in many reefs around the world (Done 1992, Bellwood et al. 2004, Hughes et al. 2007, Graham et al. 2015). Although coral–algal phase shifts are increasingly observed globally, the consequences for reef ecosystem functions such as productivity, calcification, and nutrient cycling are poorly understood. Laboratory and mesocosm studies indicate that reef algae, particularly the widespread filamentous turfs, are metabolically very different from corals, and generally display significantly higher primary production rates (Rix et al. 2015, Cardini et al. 2016). At the same time, the fraction of the photosynthetically fixed carbon (C) being exuded into the environment is generally more labile (Nelson et al. 2013). At the community level, these differences may result in changes in the carbonate chemistry of seawater (McMahon et al. 2013, Bernstein et al. 2016), disrupted trophic structures (Johnson et al. 1995, Hempson et al. 2018), or increased microbial loads on algae‐dominated reefs worldwide (Jessen et al. 2013, Haas et al. 2016).

Divergent responses to changing environmental conditions may amplify ecosystem functions of corals and algae differently. As such, changing temperature regimes and recurrent heatwaves, which are increasing in frequency and magnitude (Frölicher et al. 2018, Oliver et al. 2018), can have detrimental effects on tropical coral reef taxa (Lough et al. 2018, Hughes et al. 2019).

In corals, sublethal heat stress during summer can compromise primary production and calcification (Reynaud et al. 2003, Anthony et al. 2008), thereby altering the release of organic and inorganic products (Niggl et al. 2009, Piggot et al. 2009). In contrast, benthic turf‐ and macroalgae may be less sensitive to heat (Koch et al. 2013), showing increased productivity and net growth with rising temperature (Bender et al. 2014). Likewise, temperature‐related productivity optima and mortality thresholds of algae are often well above those of corals (Anton et al. 2020). Similarly, the abundance of reef algae can increase seasonally, especially during the summer months (Lirman and Biber 2000, Diaz‐Pulido and Garzón‐Ferreira 2002, Ateweberhan et al. 2006).

However, few studies investigated the effects of coral–algal phase shifts on community metabolism, particularly in situ. This paucity of information probably reflects the logistical challenges of quantifying the functions of structurally complex communities in their natural environment (Roth et al. 2019). Currently, most data describing ecosystem functions are derived from laboratory (e.g., Cardini et al. 2016) and mesocosm (e.g., Langdon et al. 2003, Bellworthy and Fine 2018, Edmunds et al. 2020) studies using either single organisms or simplified reconstructed communities to predict in situ changes at the community scale. Although these approaches provide valuable mechanistic insights and permit a tight control of environmental conditions during the experiments, they can only approximate natural conditions. However, primary production, calcification, and organic matter recycling critically depend on local environmental conditions, biodiversity, and system heterogeneity (Baird et al. 2007). In addition, large parts of the energy and nutrient pool are remineralized by microbial communities or cryptic fauna within the reef matrix (Richter and Wunsch 1999, de Jongh and Van Duyl 2004, Maldonado et al. 2012), all of which are generally not considered in ex situ experimental setups. Concordantly, Roth et al. (2019) highlighted in a comparison between laboratory‐based single‐organism and in situ incubations that ex situ measurements that are scaled up to average‐constructed communities can overestimate community‐wide net primary production and underestimate respiration and gross photosynthesis by 20–90%. Hence, laboratory experiments can only provide a glimpse of the complex environmental dynamics (e.g., seasonality) that shape the ecological processes of reef communities (Damgaard 2019).

To overcome these experimental constraints, we used a novel in situ approach that allowed the quantification of major metabolic and biogeochemical pathways (Roth et al. 2019) of co‐occurring natural coral‐ and algae‐dominated reef communities in the Red Sea. With a total of 112 light and dark in situ incubations, we measured rates of community production (i.e., net community production [NCP], community respiration [CR], and gross primary production [GPP]), net community calcification (NCC), net dissolved organic carbon (DOC), and dissolved inorganic nitrogen (DIN) fluxes bimonthly for an entire year. In addition, we quantified the thermal‐dependence of the functioning of benthic communities by applying principles of the metabolic theory of ecology (MTE; Sibly et al. 2012). We quantified the temperature sensitivity of metabolic processes using the activation energy (*Ea*), as the slope or rate of change in the rise and falling phases of a thermal performance curve before and after achieving the optimal temperature. Although activation energies are commonly assessed at the organism level (García et al. 2018, Savva et al. 2018, Anton et al. 2020), they also provide useful insights regarding the sensitivity of community metabolism to warming (Follstad Shah et al. 2017, Morán et al. 2017, Padfield et al. 2017).

Thus, we (1) directly compare the magnitudes and directions of key functions of coral‐dominated and phase‐shifted algae‐dominated reef communities, (2) derive their functional responses to environmental changes induced by seasonality, and (3) describe their thermal sensitivity to seasonally variable temperature changes.

## Materials and Methods

### Study site and environmental conditions

This experiment was carried out at Abu Shosha reef located in the central Red Sea on the west coast of Saudi Arabia (22°18'16.3'' N; 39°02'57.7'' E) from January 2017 until January 2018. Key environmental variables were monitored at the sampling site and were previously reported in Roth et al. (2018). Water temperature was measured continuously (logging interval = 30 min) for the whole study period with Onset HOBO temperature/light data loggers (accuracy: ±0.2°C) deployed at the seafloor. Salinity was measured at each day of sampling with a WTW TetraCon® conductivity cell (accuracy: ±0.5% of value). Light availability was measured continuously (logging interval = 1 min) on three full days per month with the above‐mentioned Onset HOBO data logger. Light readings were converted from lux to photosynthetically active radiation (PAR; μmol quanta·m^−2^·s^−1^; 400–700 nm wavelengths) by intercalibration and conversion as outlined in Roth et al. (2018), and values are presented as daytime means. Seawater samples for the determination of dissolved nitrate (${\text{NO}}_{3}^{‐}$), nitrite (${\text{NO}}_{2}^{‐}$), ammonium (${\text{NH}}_{4}^{+}$), phosphate (${\text{PO}}_{4}^{3‐}$), and monomeric silicate (Si(OH)_4_) were taken in triplicates each month from 1 m above the seafloor. Details for sampling and analysis can be found in Appendix S1: Section S1. The sum of ${\text{NO}}_{3}^{‐}$, ${\text{NO}}_{2}^{‐}$, and ${\text{NH}}_{4}^{+}$ is termed “dissolved inorganic nitrogen” (DIN) henceforth.

### Benthic communities selected for in situ incubations

Abu Shosha reef is characterized by a heterogeneous mosaic of patches of coral‐ and algae‐dominated communities. Thus, this site allows for the quantification of the functionality of both communities under identical environmental conditions. Nnatural coral‐ and algae‐dominated reef communities surrounded by sand were haphazardly selected at the study site at 5 m water depth within an area of 50 × 50 m. The communities had to fulfill the following characteristics to qualify as “suitable candidates” for later incubations: (1) Coral‐dominated communities were defined by having >40% coral cover but <10% algae cover; (2) algae‐dominated communities were defined by having >40% algae cover but <10% coral cover; (3) all communities had to fit into the incubation chambers (max. diameter 50 cm, max. height 39 cm). Among all suitable candidates in the study area, four coral‐dominated and four algae‐dominated communities were chosen randomly. These eight communities were revisited each month of sampling.

**Fig 1:**
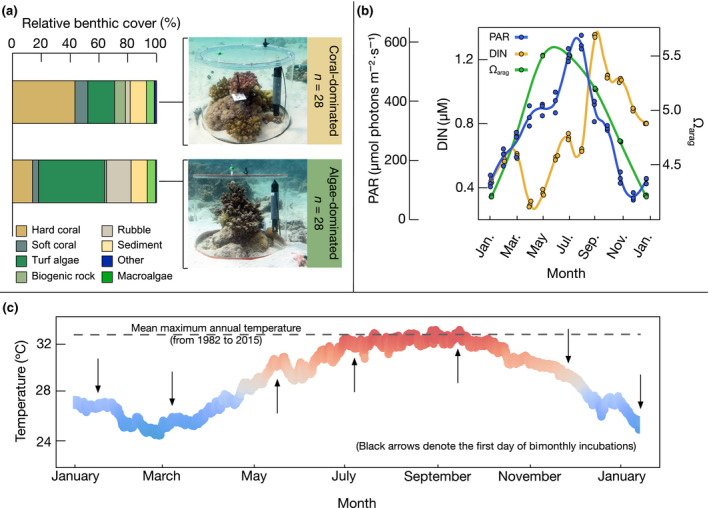
Experimental setup, relative benthic cover, and key environmental variables at the study site. (a) Relative benthic cover of functional groups in the studied coral‐ and algae‐dominated reef communities, and exemplary pictures of the incubation chambers on the respective substrates. Details on the community composition of each replicate and time point can be found in Appendix S1: Section S1 and Fig. S1. (b) Photosynthetically active radiation (PAR, in μmol photons·m^−2^·s^−1^), dissolved inorganic nitrogen (DIN, in μM), and aragonite saturation state (Ω_arag_) at the study site from January 2017 until January 2018. Circles represent values of discrete samples for DIN and Ω_arag_, and daytime averages of three separate days per month for PAR; lines represent the smoothed trend through the means. (c) Plot of seawater temperature at experimental site from January 2017 until January 2018. Each dot represents one measurement at 30‐min intervals. Dashed horizontal line depicts the mean maximum annual temperature modeled for the region from 1982 to 2015, taken from Chaidez et al. (2017).

The community composition at the level of major functional groups was assessed for each community three times during the study period (i.e., in the beginning, after 6 months, and at the end of the experiments). Details on the assessment and statistical evaluation of the community composition can be found in Appendix S1: Section S1. Two‐factor permutational multivariate analysis of variance (PERMANOVA) indicated that differences between communities grouped according to coral and algal dominance were significant (*P* = 0.001; visualization in Appendix S1: Fig. S1). As no significant changes over time in the relative benthic cover were detected (Appendix S1: Table S2),the benthic community composition of each treatment was averaged over all replicates and survey points (Fig. 1a).

Algae‐dominated communities were considered “phase‐shifted,” as complex structures and the occurrence of coral rubble indicated that branching corals were present previously. The co‐occurrence of coral and algal reef communities within small spatial ranges was reported as “mosaic‐dynamics” before (e.g., Edmunds 2002, Tkachenko et al. 2007) and may be explained by a combination of local processes and historical effects, such as previous stress events or adaptation (Done et al. 1991, Bythell et al. 2000, Edmunds 2002).

Cryptic habitats can encompass about 60–75% of the total surface area of a reef (Richter and Wunsch 1999, Richter et al. 2001), but organisms living in cracks and crevices within the communities’ matrices could not be assessed by our conventional benthic surveys. These organisms (e.g., sponges, bryozoans, and tunicates), however, metabolize organic matter in the order of 15–30% of the gross production of a coral reef (reviewed in de Jongh and Van Duyl 2004), driving community respiration and other biogeochemical fluxes assessed in this study. As our benthic incubation chambers jointly captured the metabolism of all members of the communities (i.e., from the visible surface and cryptic habitats), we refrained from assigning measured metabolic activities to functional groups on the visible surface only, as the inferred contribution would be highly biased. Thus, measurements presented in this study represent community‐wide processes that include all compartments of the reef benthos and the surrounding water.

### In situ incubations and quantification of community functions

In situ incubations with benthic chambers were performed according to the protocol described in Roth et al. (2019). In brief, chambers were constructed from polymethyl methacrylate (PMMA) cylinders with removable gastight lids of the same material. All chambers were equipped with individual water circulation pumps with adjustable flow control, autonomous recording dissolved oxygen (DO), and temperature sensors (HOBO U26; temperature corrected and salinity adjusted), and two sampling ports for discrete water samples. Incubations were carried out on three consecutive days in January, March, May, July, September, and November 2017, and in January 2018. Generally, on day 1, divers deployed four chambers on coral‐dominated, and four chambers on algae‐dominated reef communities (Fig. 1a). The chambers were positioned carefully and left in place with open tops (no lids) until the next morning. On the second day, incubations started at around 09:00 a.m. by tightly securing the lids and closing all sampling ports during natural daylight conditions. The exact incubation start and end time was recorded for each chamber. Incubations ran for approximately 2 h. The chambers were left in place with open tops for a second set of incubation on the following day. On day 3, benthic communities were incubated at “simulated” darkness during the same period used for incubations the previous day. The procedure followed the same as on day two; however, all chambers were covered with thick black PVC covers. Any light penetration into the chambers was prevented, as validated by control readings of Onset HOBO temperature/light data loggers within chambers. Between deployments, all materials were rinsed with freshwater, washed with 4% hydrochloric acid (HCl), and subsequently rinsed with deionized water for reliable water chemistry measurements that included sensitive DOC samples.

Discrete water samples for dissolved inorganic carbon (DIC), total alkalinity (TA), DOC, and DIN were withdrawn from the sampling ports with acid‐washed syringes at the beginning and the end of each incubation (details for the analysis of these samples can be found in Appendix S1: Section S1). Changes in seawater chemistry between start and end of incubations were used to calculate rates of NCP, CR, GPP (calculated as GPP = NCP + |CR|), NCC, and fluxes of DOC, and DIN. All rates and fluxes were extrapolated to incubation water volume (in L) and normalized to incubation duration (in h) and the planar reef area (in m^2^) of the enclosed benthic community adapted after Roth et al. (2019).

Productivity and respiration rates (NCP and CR; in mmol C·m^−2^·h^−1^) were calculated by changes in DIC concentrations, taking into account calcification and dissolution rates according to an adapted protocol by Albright et al. (2013). Rates of NCP and CR based on DIC fluxes were compared to rates based on oxygen fluxes from continuous measurements with DO sensors. No discrepancy between C and oxygen measurements was detected (*r* = 0.99, *P* < 0.0001, *n* = 112). The calculated photosynthetic (1.05 ± 0.02) and respiratory (0.97 ± 0.02) quotients agree with those obtained by various authors elsewhere, typically ∼1 mol of oxygen produced for 1 mol C fixed, and vice versa (e.g., Gattuso et al. 1999b, Atkinson and Falter 2003).

NCC (in mmol CaCO_3_·m^−2^·h^−1^) was calculated by concentration differences in TA, which are primarily caused by calcification and dissolution of CaCO_3_, whereby TA is reduced (increased) by two molar equivalents for every mole of CaCO_3_ produced (dissolved) (Zeebe and Wolf‐Gladrow 2001). Nutrients fluxes (i.e., ${\text{NO}}_{3}^{‐}$, ${\text{NH}}_{4}^{+}$, ${\text{PO}}_{4}^{3‐}$, and ${\text{SO}}_{4}^{2‐}$) that cause a change in TA unrelated to calcification and dissolution were accounted for according to Zeebe and Wolf‐Gladrow (2001) and Wolf‐Gladrow et al. (2007). DOC (in mmol C·m^−2^·h^−1^) and DIN (in µmol N·m^−2^·h^−1^) fluxes were calculated from concentration differences between start and endpoints. Any temperature corrections that were necessary for seawater chemistry calculations were achieved by temperature readings from individual temperature loggers within each chamber.

Although measurements presented in this study only relate to small benthic communities, most studies currently work with individual reef organisms (e.g., Anton et al. 2020) or reconstructed communities (e.g., Edmunds et al. 2020) to derive community functions. Thus, the results presented here are among the best approximations for the quantification of community‐wide (biogeochemical) ecosystem functions of untouched, natural coral reef communities in situ (but see Haas et al. 2013, Van Heuven et al. 2018).

### Data analysis

Statistical analyses were performed using JMP© Pro14 (SAS Institute) statistic software. Environmental variables and response parameters from incubations were grouped into spring (March–May), summer (June–September), fall (October–November), and winter (December–February) for statistical analysis. For the seasonal comparison and to derive GPP/CR ratios, hourly rates from light and dark incubations were used to calculate daily net fluxes. We acknowledge that there is a chance for a slight over‐ or underestimation because of associated changes in environmental conditions (e.g., light) during the course of the day. Thus, to minimize the error associated with extrapolating hourly rates, we chose a time window for daylight incubations (from around 09:00 a.m. to 11:00 a.m.) that is closest to daytime average irradiation and excludes the “ramping up” phase in the early morning hours and extreme values that can occur during midday. As all incubations during all sampling periods were conducted at the very same time of the day, incubations are comparable across community types and time points.

The full seawater carbonate system parameters were derived for each sampling period from measured salinity, temperature, nutrients, TA, and DIC data using the CO2SYS Microsoft Excel Macro by Pierrot et al. (2006) and the R package Seacarb (Lavigne and Gattuso 2013) (Appendix S1: Table S3). Environmental variables were tested for differences with two‐tailed *t*‐tests. Response parameters from incubations (GPP, NCP, CR, NCC, DOC, and DIN) were assessed by linear mixed models (LMMs) to test for differences in the respective response parameters with ‘treatment’ (coral‐ vs. algae‐dominated) and ‘season’ (spring, summer, fall, and winter) as fixed factors, and the sampling dates (date) within seasons and the replicates of the communities (community ID) as random factors. Tukey’s Honest Significant Difference (HSD) test was used for pairwise comparisons if significant interactions (treatment * season) were found. Detailed statistical results, including significant post hoc comparisons, are presented in Appendix S1: Table S4. The relationships between response (e.g., metabolic functions) and explanatory variables (e.g., environmental variables) were assessed by linear regression models.

The thermal sensitivity of the metabolic processes (GPP, NCP, CR, NCC, DOC, and DIN) was explored by calculating the activation energy (*Ea*) based on Arrhenius equations (Sibly et al. 2012) within the seasonal thermal regime (25.0–32.8°C). The activation energies (*Ea* in eV) were estimated by fitting a linear regression equation between the natural logarithm of the metabolic rates and the reciprocal of temperature (1/*kT*), where *k* is the Boltzmann’s constant (8.62 × 10^−5^ eV/K) and *T* is the water temperature (K). To deal with observations ≤0 on log‐transformed data (e.g., NCC, DOC, and DIN rates), a constant was added (i.e., ln(rate + 1 − min value(rate)) to shift all values above zero (Legendre and Legendre 2012). The alternative of excluding ≤0 values was discarded because these measurements are an important part of the biological processes under investigation (Canavero et al. 2018).

## Results

### Environmental conditions

Monthly monitored environmental variables at the study site exhibited strong seasonal patterns (Fig. 1b,c, Appendix S1: Table S2). The average seawater temperature ranged from 25.8 ± 0.2°C in winter to 32.3 ± 0.1°C in summer (Fig. 1c). Simultaneously, average daytime PAR intensities at 5 m water depth increased from 130 ± 2 μmol photons·m^−2^·s^−1^ to 465 ± 14 μmol photons·m^−2^·s^−1^ (Fig. 1b). Seawater DIN concentrations were lowest in spring and winter (0.46 ± 0.02 and 0.66 ± 0.04 μM DIN, respectively) and significantly higher in summer and fall (1.03 ± 0.06 and 1.08 ± 0.13 μM DIN, respectively, Fig. 1b).

### Functions of coral‐ and algae‐dominated reef communities

**Fig 2:**
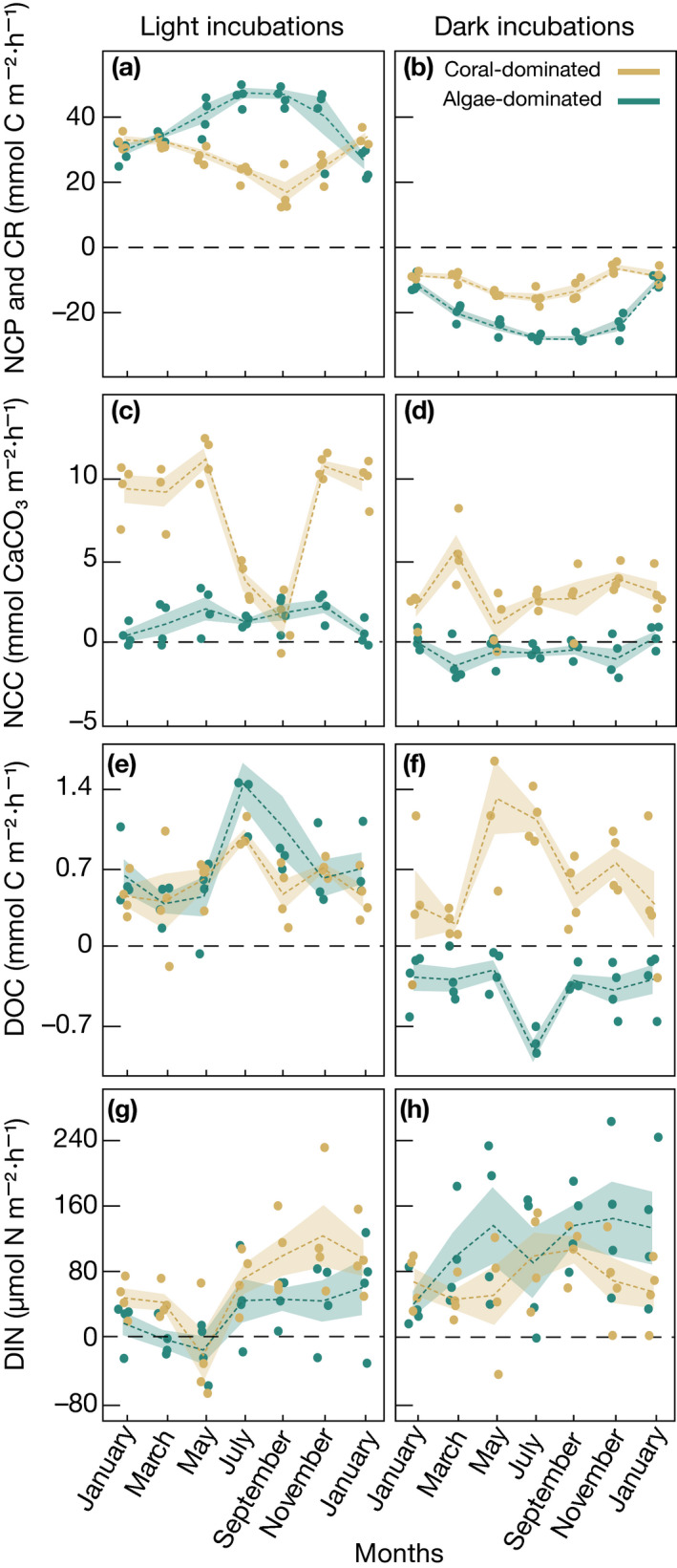
Hourly biogeochemical fluxes during light (left panels) and dark (right panels) incubations of coral‐ and algae‐dominated reef communities of the central Red Sea. Presented are all data from bimonthly incubations of (a, b) community metabolism (divided into net community production (NCP) and community respiration (CR), (c, d) net community calcification (NCC), (e, f) net dissolved organic carbon (DOC) fluxes, and (g, h) net dissolved inorganic nitrogen (DIN) fluxes. Dashed lines connect the bimonthly means. Shaded areas connect the standard error around the means.

The rates observed along the various deployments (Fig. 2) were used to calculate average values over the whole study period (Table 1). Average NCP was 30% and CR 50% lower in coral‐ as compared to algae‐dominated communities (NCP, mean ± SE: 26.7 ± 1.2 and 36.9 ± 1.7 mmol C·m^−2^·h^−1^, respectively; CR: −10.9 ± 0.7 and −20.9 ± 1.4 mmol C·m^−2^·h^−1^, respectively; Fig. 2a,b). Integrated over 1 d, these differences yielded a 40% lower GPP of coral‐ compared to algae‐dominated communities, with GPP/CR ratios of 2.4 ± 0.1 and 2.0 ± 0.2, respectively (Table 1).

**Table 1:** Seasonal gross primary production (GPP), net community production (NCP), community respiration (CR), the ratio of GPP and CR (GPP/CR), net community calcification (NCC), net dissolved organic carbon (DOC) fluxes, and net dissolved inorganic nitrogen (DIN) fluxes of coral‐ and algae‐dominated reef communities of the central Red Sea.

	GPP (mmol C⋅m⁻^2^⋅d⁻^1^)	NCP (mmol C⋅m⁻^2^⋅d⁻^1^)	CR (mmol C⋅m⁻^2^⋅d⁻^1^)		GPP/CR	NCC (mmol CaCO₃⋅m⁻^2^⋅d⁻^1^)	DOC (mmol C⋅m⁻^2^⋅d⁻^1^)	DIN (mmol N⋅m⁻^2^⋅d⁻^1^)
Annual mean								
Coral	581 ± 19	320 ± 15	−261 ± 17		2.4 ± 0.1	131 ± 10	14.3 ± 1.8	1.6 ± 0.2
Algae	946 ± 51	443 ± 21	−503 ± 33		2.0 ± 0.2	10 ± 3	4.0 ± 0.9	1.7 ± 0.2
*P*	<0.0001*	<0.0001*	<0.0001*		<0.0001*	<0.0001*	<0.00001*	0.9351
Spring								
Coral	638 ± 21	353 ± 12	−285 ± 25		2.3 ± 0.2	162 ± 11	14.1 ± 3.6	0.7 ± 0.4
Algae	974 ± 41	439 ± 22	−535 ± 27		1.8 ± 0.0	7 ± 6	1.6 ± 1.6	1.3 ± 0.4
∣*t*∣	<0.0001*	0.0833	<0.0001*		0.0134*	<0.0001*	0.0246*	0.9573
Summer								
Coral	575 ± 45	231 ± 24		−344 ± 26	1.7 ± 0.1	61 ± 11	18.9 ± 3.9	2.2 ± 0.2
Algae	1,224 ± 13	553 ± 12		−671 ± 9	1.8 ± 0.0	13 ± 3	7.1 ± 1.9	1.9 ± 0.4
∣*t*∣	<0.0001*	<0.0001*		<0.0001*	0.9310	0.0024	0.0432*	0.9979
Fall								
Coral	447 ± 44	292 ± 25		−154 ± 20	2.9 ± 0.1	176 ± 7	16.2 ± 1.7	2.3 ± 0.7
Algae	1,053 ± 94	470 ± 68		−583 ± 43	1.8 ± 0.1	15 ± 11	2.3 ± 3.0	2.3 ± 0.7
∣*t*∣	<0.0001*	0.0017*		<0.0001*	<.00001*	<0.0001*	0.0111*	1.0000
Winter								
Coral	597 ± 20	390 ± 10		−207 ± 15	2.9 ± 0.1	147 ± 10	9.1 ± 3.0	1.6 ± 0.3
Algae	586 ± 30	324 ± 17		−262 ± 18	2.3 ± 0.1	8 ± 4	4.1 ± 0.4	1.5 ± 0.5
∣*t*∣	1.0000	0.3351	0.6166		0.0005	<0.0001*	0.1388	1.0000

Notes

Values represent averages from all incubations during the respective season ± SE. Significant differences between treatments were assessed by linear mixed models (LMMs), and the differences between treatments and seasons by Tukey’s Honest Significant Difference (HSD; Appendix S1: Table S4) test. Asterisks highlight significant *P* values (*P* or |*t*| < 0.05).

NCC in the light was sixfold higher in coral‐ compared to algae‐dominated communities, averaging 7.9 ± 0.7 mmol and 1.3 ± 0.2 mmol CaCO_3_·m^−2^·h^−1^, respectively. In the dark, coral‐dominated communities displayed an NCC of 3.0 ± 0.3 mmol CaCO_3_·m^−2^·h^−1^, whereas algae‐dominated communities exhibited an NCC of −0.5 ± 0.2 mmol CaCO3·m^−2^·h^−1^ (representing net CaCO_3_ dissolution). Integrating the hourly rates over 24 h, corals showed a 10‐fold higher NCC compared to algae‐dominated reef communities (Table 1).

Coral‐dominated communities were net sources of DOC during both light and dark incubations, with average fluxes of 0.57 ± 0.07 and 0.62 ± 0.11 mmol C·m^−2^·h^−1^, respectively (Table 1). In contrast, algae‐dominated communities released similar amounts of DOC as corals in the light (0.70 ± 0.08 mmol C·m^−2^·h^−1^) but were net sinks of DOC in the dark (−0.37 ± 0.05 mmol C·m^−2^·h^−1^). When integrated over 24 h, net DOC fluxes in coral‐dominated communities were 3.5‐fold higher than those in algae‐dominated communities (Table 1).

Both coral‐ and algae‐dominated communities were net sources of DIN (1.62 ± 0.21 and 1.66 ± 0.23 mmol N·m^−2^·d^−1^, respectively; Table 1), with no significant differences between treatments. There were, however, significant differences between light and dark incubations: algae‐dominated communities released three times more DIN in the dark as compared to light conditions (109.9 ± 14.1 μmol N·m^−2^·h^−1^ and 28.5 ± 8.7, respectively). In contrast, coral‐dominated communities released DIN at consistent rates during light and in dark incubations (65.7 ± 11.6 and 69.7 ± 8.9 μmol N·m^−2^·h^−1^).

### Temporal variability of reef functions

Both C and N fluxes showed temporal variations; however, significant differences in the magnitude and directions occurred between coral‐ and algae‐dominated reef communities (Fig. 2, Table 1; detailed statistics in Appendix S1: Table S4).

**Fig 3:**
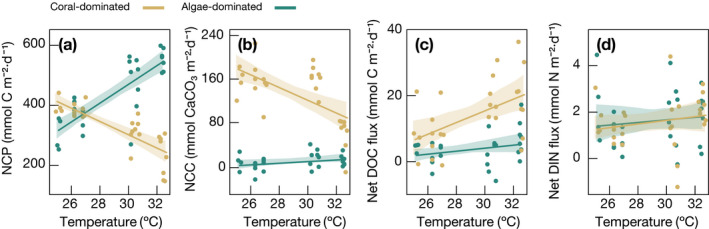
Relationship of temperature with (a) net community production (NCP), (b) net community calcification (NCC), (c) dissolved organic carbon (DOC) fluxes, and (d) dissolved inorganic nitrogen (DIN) fluxes in coral‐ and algae‐dominated reef communities. Rates represent the net flux integrated over a 24‐h day (assuming 12 h of light and 12 h of dark). Solid lines represent the linear regressions; shaded areas in transparent colors represent the 95% confidence intervals.

Daily‐integrated GPP in coral‐dominated communities remained stable throughout the year; however, CR increased by >60% from winter to summer, resulting in 40% lower NCP (Table 1). GPP in algae‐dominated communities doubled from winter to summer, resulting in significantly increased NCP that peaked at >500 mmol C·m^−2^·d^−1^ in summer. In both community types, variations in NCP were significantly correlated with seawater temperature (Fig. 3a). NCP of coral‐dominated communities exhibited a negative (*r* = −0.81, *P* < 0.0001, *n* = 26), and NCP of algae‐dominated communities exhibited a positive (*r* = 0.83, *P* < 0.0001, *n* = 26) relationship with increasing temperature. Hence, coral‐dominated communities showed apparent negative *Ea* values for GPP and NCP, indicating them to be in the falling phase of the performance curve (i.e., past the optimum temperature), while apparent *Ea* for CR was positive, indicating an opposite trend (Appendix S1: Fig. S3). In contrast, algae‐dominated communities had positive *Ea* values for GPP, NCP, and, CR (Table 2), indicating that these communities remained within the rising phase of the performance curve throughout the study period (including the summer months; Appendix S1: Fig. S3).

**Table 2:** Apparent activation energies (*Ea*, eV) for coral‐ and algae‐dominated reef communities as the slope of the Arrhenius relationship between the natural logarithm of specific metabolic rates and the inverted temperature (1/*kT*).

	GPP		NCP		CR		NCC		DOC		DIN	
	Coral	Algae	Coral	Algae	Coral	Algae	Coral	Algae	Coral	Algae	Coral	Algae
*Ea* (eV)	−0.09	0.75	−0.58	0.55	0.49	0.93	−0.82	0.65	0.73	0.19	0.12	0.13
*r* ^2^	0.03	0.71	0.54	0.59	0.26	0.66	0.30	0.10	0.28	0.01	0.02	0.02
*P*	0.3861	<0.0001	<0.0001	<0.0001	0.0065	<0.0001	0.0023	0.1073	0.0038	0.5619	0.5126	0.4439

Notes

GPP = gross primary production (mmol C·m^−2^·d^−1^); NCP = net community production (mmol C·m^−2^·d^−1^); CR = community respiration (mmol C·m^−2^·d^−1^); NCC = net community calcification = (mmol CaCO_3_·m^−2^·d^−1^); DOC = dissolved organic carbon fluxes (mmol C·m^−2^·d^−1^); DIN = dissolved inorganic nitrogen fluxes (mmol N·m^−2^·d^−1^); *r* ^2^ = square of correlation coefficient.

**Fig 4:**
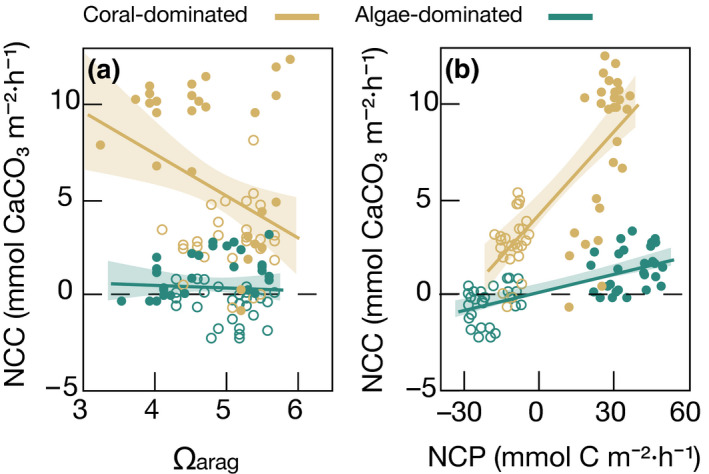
Relationship between (a) net community calcification (NCC) and aragonite saturation state (Ω_arag_), and (b) NCC and net community production (NCP) in coral‐ and algae‐dominated reef communities. Closed circles indicate measurements from light incubations, and open circles indicate dark incubations. Solid lines represent the linear regressions; shaded areas in transparent colors represent the 95% confidence intervals.

NCC in coral‐dominated communities peaked in spring, fall, and winter, with no differences between these seasons, but dropped sharply by >60% in summer (Table 1). Contrary, NCC in algae‐dominated communities was consistently low throughout the year (Fig. 2c,d; Table 1). NCC in coral‐dominated communities correlated negatively with increasing water temperatures (*r* = −0.62, *P* = 0.0005, *n* = 26; Fig. 3b), negatively with Ω_arag_ (*r* = −0.36, *P* = 0.0070, *n* = 54) (Fig. 4a), and positively with NCP (*r* = 0.73, *P* < 0.0001, *n* = 54; Fig. 4b). NCC of algae‐dominated communities did neither correlate significantly with Ω_arag_, NCP, nor water temperature. The corresponding apparent *Ea* values are presented in Table 2.

Net DOC fluxes per day in coral‐dominated communities were lowest in winter and doubled in summer (Table 1), owing to both increases in DOC releases during dark and light incubations. While generally one order of magnitude lower, net DOC fluxes integrated per day remained stable in algae‐dominated communities throughout most of the year but increased towards summer (Table 1). Both community types showed a positive correlation between net DOC fluxes and increasing temperature (Fig. 3c), which was also reflected in positive *Ea* values (Table 2, Appendix S1: Fig. S3).

DIN fluxes in both coral‐ and algae‐dominated communities were lowest in spring and twofold higher during the rest of the year (Table 1). DIN fluctuations did not correspond to changes in seawater temperature (Fig. 4d) but showed a significant positive relationship (*r* = 0.57, *P* = 0.0017, *n* = 26 for corals; *r* = 0.39, *P* = 0.0385, *n* = 26 for algae) with increasing DIN concentrations of the ambient seawater (Appendix S1: Fig. S2).

## Discussion

### Phase shifts from corals to algae may drastically change reef community functions

The carbon and carbonate pathways are well described in the literature for undisturbed coral reef communities, both in direction and magnitude (Appendix S1: Table S5). Concomitant to previous findings, coral‐dominated communities in our experiments displayed: (1) low net community production despite high gross productivity, implying that biomass accumulates slowly (Gattuso et al. 1998); (2) net DOC fluxes, where the accumulation of exudates outpaces its consumption (Nelson et al. 2013, Quinlan et al. 2018), representing an organic C loss that further reduces C available to support net benthic biomass accretion, and promotes an efficient transfer of C via benthic‐pelagic coupling (Wild et al. 2004); and (3) high rates of net community calcification, consistent with the accretion of carbonate structures typical for tropical coral reefs (Gattuso et al. 1998, 1999b, Atkinson and Falter 2003). Yet, our seasonal comparative in situ approach revealed that functions of reef ecosystems change with shifts from a coral‐ to an algae‐dominated benthic community.

Specifically, algae‐dominated communities displayed a higher organic C metabolism, along with residual net calcification. Significantly higher GPP and >40% elevated NCP in algae‐dominated communities indicate a greater potential for autotrophic biomass accumulation per planar square meter of reef (Fong and Paul 2011, Kelly et al. 2017), despite a lower GPP/CR ratio (Table 1). The lower GPP/CR ratio in algae communities compared to coral communities seems counterintuitive considering the dominant organisms (algae as autotrophs compared to mixotrophic corals); however, algae‐dominated reef structures host numerous heterotrophs that rely on the high algal biomass production, fueling community‐wide respiration by direct herbivory (Klumpp and McKinnon 1989, Russ 2003) and indirect detritivory by invertebrates (Kramer et al. 2013). Likewise, sponges and other filter feeders are commonly associated with degraded reef habitats (Abele and Patton 1976), feeding on DOC or algal debris (Rix et al. 2017, 2018). In addition, heterotrophic bacteria within algal communities remineralize labile DOC released by algae (Nelson et al. 2011, Haas et al. 2013). Thereby, the consumption of algal‐derived C (i.e., the C retention within the system) can shorten the average trophic path length, and reduce the average trophic level of the second‐order consumers (reviewed in Johnson et al. 1995). In support of these trophic interactions, we observed an apparent consumption of DOC in the dark in algae‐dominated communities, limiting the net DOC flux integrated over 24 h (i.e., release in the light balanced by consumption in the dark). Our results indicate that algae‐associated organisms readily remineralize DOC, concurring with demonstrated DOC depletion in algae‐dominated shallow reefs elsewhere (Nelson et al. 2011, Haas et al. 2016).

Along with alterations of the organic C cycle, calcification was reduced within algae‐dominated communities. The slope of the relationship between NCP and NCC is commonly used as an indicator of reef health, indicative for the relative proportion of calcifying to noncalcifying organisms in a benthic community (Albright et al. 2013, Takeshita et al. 2016). We observed a slope of 0.18 in coral‐ and 0.03 in algae‐dominated communities, and the slope averages 0.22 based on 52 reefs around the world (Gattuso et al. 1999a). The significant difference highlights the shift from calcifying corals to noncalcifying organisms and a decoupling of the organic carbon (production vs. respiration) and carbonate (calcification vs. dissolution) cycles in algae‐dominated communities (McMahon et al. 2019).

Unraveling nitrogen pathways in coral reefs is crucial to understand how high productivity is supported despite low ambient nutrient concentrations (D’Elia and Wiebe 1990, Szmant 2002, Atkinson and Falter 2003). Both coral‐ and algae‐dominated communities were net sources of DIN over the study period with no significant differences between community types. The flux rates generally fell within the published range of in situ measurements (−4 to 5 mmol N as NO_x_·m^−2^·d^−1^; reviewed in Atkinson and Falter 2003). They are in stark contrast, however, with the expectation that net autotrophic communities would act as sinks for dissolved inorganic nutrients, as generally measured in single organism incubations (e.g., Den Haan et al. 2016). Although a theoretical N requirement of 34–83 mmol N·m^−2^·d^−1^ to support NCP can be expected (stoichiometric calculations with NCP ranging from 230 to 550 mmol C·m^−2^·d^−1^; assuming a C/N ratio of 6.6; see Redfield 1958), the effects of assimilation were likely masked by concurrent community‐wide processes that produce DIN (Gruber et al. 2019). For example, cavities within Red Sea reefs can be considerable sources of DIN (>20 mmol N·m^−2^·d^−1^) as sponges and other filter feeders utilize dissolved organic matter (Richter et al. 2001). Also, microbial communities can consume and transform organic N compounds (Yahel et al. 2003, Moulton et al. 2016, Pfister and Altabet 2019), potentially increasing the community‐wide DIN release into the environment. Other pathways, such as N_2_ fixation (Cardini et al. 2016) or heterotrophic feeding on particulates (Ribes et al. 2003, Houlbrèque and Ferrier‐Pagès 2009) are additional N sources that potentially limit/mask the N uptake from DIN.

Overall, algae‐dominated communities displayed a higher potential of biomass accumulation or export (i.e., high NCP), associated with a higher total amount of C available (i.e., high GPP) to the ecosystem. The high NCP of algae communities facilitates rapid lateral and vegetative overgrowth of bare substrates (Diaz‐Pulido and Garzón‐Ferreira 2002, Roth et al. 2018). At the same time, limited reef accretion (i.e., low NCC) within algal habitats may compromise the topographic complexity of phase‐shifted reef communities (Wild et al. 2011), limit the recruitment of corals (Harrington et al. 2004, Roth et al. 2017, 2018), and increase reef erosion (Adey 1978).

### High temperatures during summer amplify functional differences between coral‐ and algae‐dominated communities

**Fig 5:**
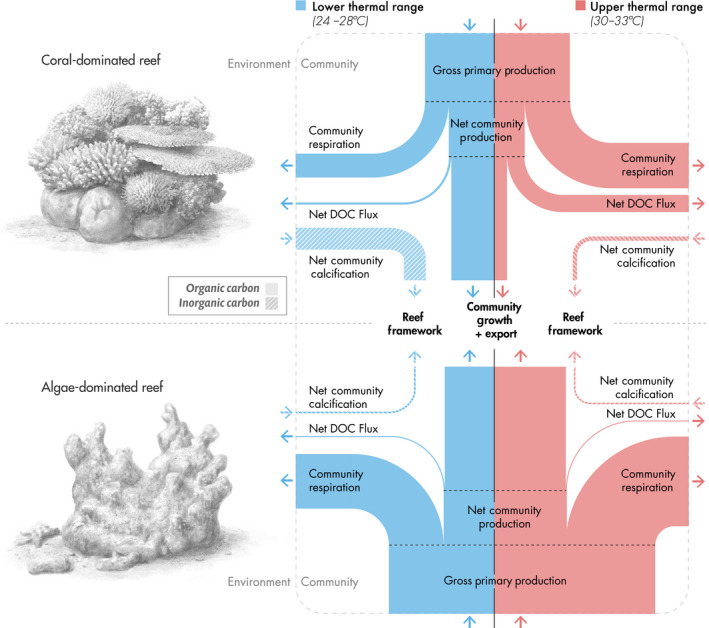
In situ community metabolism of natural coral‐ and algae‐dominated reef communities in the central Red Sea, Saudi Arabia. Schematic was derived from all data available in the given lower (blue) and upper (red) temperature ranges. Organic carbon pathways refer to photosynthesis, respiration, and dissolved organic carbon (DOC) fluxes, and the inorganic carbon pathway refers to the formation and dissolution of calcium carbonate. The thicknesses of the bars scale with the actual flux measurements from in situ incubations.

Our data highlight that functions related to the carbon and carbonate cycles of coral‐ and algae‐dominated communities are strongly but inversely affected by temperature (summarized in Fig. 5), with implications for their response to warming.

The results from activation energies show a higher sensitivity to thermal stress of coral‐dominated compared to algae‐dominated communities during summer (Table 2, Appendix S1: Fig. S3). Particularly, apparent activation energies for NCP and NCC of coral‐dominated communities (*Ea* = −0.58 and −0.82 eV; respectively) were in the falling phase of the performance curves, and thus, past the optimum temperature to peak rates. Thereby, the community metabolism of corals is pushed toward carbon losses because of high CR relative to GPP in summer. Likewise, the activation energy of CR was positive, as respiration continued to increase with temperature through the seasonal thermal range. Both the lower ratio of GPP to CR (GPP/CR) and reduced NCP integrated over diel cycles indicate that coral‐dominated communities shifted toward a more heterotrophic state with increasing temperature. It is thus apparent that summer temperatures exceeded the metabolic optima for coral‐dominated communities, which was previously suggested for individuals of *Pocillopora verrucosa* (Sawall et al. 2015, Roik et al. 2016, Anton et al. 2020) and *Stylophora pistillata* (Anton et al. 2020) in the central Red Sea. This contrasts with many reef locations worldwide, where primary production maxima are typically observed during the warmest months of the year (e.g., Scheufen et al. 2017). At the same time, coral‐dominated communities displayed enhanced rates of net DOC fluxes with warming (*Ea* = 0.73 eV), which can be attributed to an increased release of cellular matter and/or mucoid exudates during thermal stress in corals (Niggl et al. 2009, Scheufen et al. 2017). Although mucus released during higher temperatures may help to protect corals against pathogens (Glasl et al. 2016) or high UV radiation (Gleason and Wellington 1993), it poses an increased loss of organic C that can be used for community growth and/or export at constant GPP rates (Fig. 5). Along with this trend, NCC dropped by >50% from the annual mean during summer, with most of this decline realized as waters warmed from 30 to 32°C. Overall, decreased NCC strongly correlated with decreased NCP as temperatures increased (as revealed by high negative apparent activation energies of NCC, *Ea* = −0.82 eV), with a temperature threshold at around 30.5°C (Appendix S1: Fig. S3). This thermal threshold is near the reported thermal optimum of *Pocillopora verrucosa* and *Stylophora pistillata* for gross primary production (29.9 and 31.9°C, respectively; Anton et al. 2020) in the central Red Sea, indicating a strong thermal sensitivity of coral‐dominated communities soon after the thermal optimum is exceeded. Accordingly, in the temperature range above the optimum, the rate of calcification decreased despite increased Ω_arag_ in summer (Fig. 4a; Silverman et al. 2007). Physiological factors can also strongly affect the biomineralization process. As calcification mainly depends on the photosynthetic efficiency of the endosymbionts within corals (Gattuso et al. 1999a, Allemand et al. 2004), a lower NCC may occur for thermally stressed corals, limiting reef accretion and stabilization (Jokiel and Coles 1977, De’ath et al. 2009).

In contrast, positive activation energies for algae‐dominated communities (*Ea* = 0.93, 0.75, and 0.55 eV for CR, GPP, and NCP; respectively) indicate that these communities benefit from higher temperatures and that thermal optima were not reached in summer. In fact, some macroalgae species (*Halimeda tuna*) from the central Red Sea have a reported thermal optimum (31.7°C) for gross primary production (Anton et al. 2020) that is close to the maximum summer temperature recorded during our incubations (32.5°C). The increases in GPP and CR along a thermal gradient in algae‐dominated communities highlight a higher turnover of organic C. However, increases in C fixation outweighed increases in respiratory C consumption, resulting in higher NCP in the summer. In contrast to previous reports (e.g., Barron et al. 2014), DOC fluxes in algae‐dominated communities showed only a weak temperature dependence. However, although the overall net DOC fluxes remained relatively stable, differences in net production during the light and net consumption in the dark amplified with temperature (Fig. 3), limiting losses of organic C through this process. As a result, more organic C was retained within algae‐dominated communities in summer, supporting biomass accumulation and export in the community (Fig. 5).

### Implications for reef ecosystem functioning under global change

Theoretical studies have provided a sound understanding of the relationship between biodiversity loss and ecosystem functions in tropical coral reefs (reviewed in Hughes et al. 2017). However, considerable knowledge gaps remain, in particular, on how metabolic and biogeochemical processes differ between coral‐ and algae‐dominated communities, and how these respond to seasonal fluctuations in environmental conditions. As algal cover is expected to increase in coral reefs, our long‐term in situ experiments reveal how these novel communities in general, and how thermal stress in particular, may alter pivotal ecological functions of future reefs. Our data show that fundamental metabolic and biogeochemical characteristics of coral‐dominated communities are disturbed by shifts from coral to algal dominance and may, thereby, compromise the future stability and resilience of coral reef biota. These responses may be further compounded by differential thermal responses between coral and algae species (e.g., Anton et al. 2020).

The sensitivity of corals and their symbionts to rising temperatures has been documented extensively (Hoegh‐Guldberg 1999). Thermal anomalies exceeding 1–2°C above the mean summer maximum temperature can compromise the symbiosis (e.g., Weeks et al. 2008), leading to coral bleaching and reduced coral survival (Baird and Marshall 2002, Baker et al. 2008). However, our study did not record temperatures exceeding the local mean summer maxima reported for the region (see Fig. 1c; Chaidez et al. 2017) and, likewise, no apparent signs of coral bleaching were observed. Nevertheless, growth of *Pocillopora verrucosa* and *Stylophora pistillata* (Anton et al. 2020) and calcification of *Pocillopora verrucosa* (Roik et al. 2018) are already reduced under current summer conditions in the Red Sea, as also highlighted by the present study. For the warmer part of the year, algae‐dominated communities have, thus, a metabolic advantage over coral‐dominated communities because they maintain high NCP. Importantly, the consequences of global warming may manifest not only in terms of higher‐than‐normal temperatures but also in a longer‐than‐normal duration of the seasonal peak temperatures (Fitt et al. 2001). Under such conditions, if coral mortality events occur, algae may quickly spread and shift coral ecosystems more rapidly towards systems that are dominated by algae (McManus et al. 2019), as already reported on some reefs in the southern Red Sea following the coral bleaching event in 2015 (Anton et al. 2020).

The frequency and intensity of climate‐driven stress events on coral reefs will inevitably aggravate in the near future. Our results suggest that the anticipated increase in the spatial footprint of algae‐dominated communities would exacerbate the magnitude of the functional changes described here. Ocean warming likely enhances the competitive advantage of algae‐ over coral‐dominated communities (Anton et al. 2020), thus promoting a positive feedback loop of reef degradation. Similar effects of warming are likely to be operational in other temperature‐sensitive and calcifying communities. As these organisms are central to the formation of reef ecosystems, critical changes in the biodiversity and functioning may be witnessed. Thus, appropriate management practices designed to limit the proliferation of algae are needed for maintaining reefs dominated by corals and the important ecosystem services they support.

## Supporting information

Appendix S1
