# Supplementary material for: High summer temperatures amplify functional differences between coral‐ and algae‐dominated reef communities
Source: Ecology. 2020 Dec 27;102(2):e03226. doi: 10.1002/ecy.3226 (PMC7900985; doi:10.1002/ecy.3226)
Supplement: Supplementary file 1 — Appendix S1 [file ECY-102-e03226-s001.pdf]

Supporting Information. Roth, F., N. Rädecker, S. Carvalho, C.M. Duarte, V. Saderne, A. Anton, L. Silva, M.Ll. Calleja, X.A.G. Morán, C.R. Voolstra, B. Kürten, B.H. Jones, and C. Wild. 2020. High summer temperatures amplify functional differences between coral- and algae-dominated reef communities. Ecology.

Appendix S1

Section S1: Water sampling and analysis

For the characterization of environmental background conditions, seawater samples for the determination of dissolved nitrate (NO_3^-), nitrite (NO_2^-), ammonium (NH_4^+), phosphate (PO_4^{3-}), and monomeric silicate ($Si(OH)_4$) were taken in triplicates each month from 1 m above the seafloor with 60 mL acid-washed syringes. On the boat, samples were filtered immediately through syringe filters (IsoporeTM membrane filters, 0.2 µm GTTP) into acid-washed 15 mL centrifuge tubes and stored dark and cool for transportation to the laboratory (< 2 h). In the laboratory, samples were stored frozen at − 50°C pending analysis using a continuous flow analyzer (AA3 HR, SEAL, USA) following the designated colorimetric methods (Grasshoff et al. 1999). The limits of quantification (LOQ) were 0.084 µM NO_3^-, 0.011 µM NO_2^-, 0.043 µM PO_4^{3-}, and 0.191 µM $Si(OH)_4$, respectively. From the syringes, 5 mL subsamples were filtered separately into acid-washed 15 mL centrifuge tubes for ammonium (NH_4^+) measurements underway using the ortho-phthaldialdehyde (OPA) method (Holmes et al. 1999, Taylor et al. 2007). To the filtered sample, 1.2 mL OPA-solution was added and NH_4^+ was determined fluorometrically within 8 h after sampling (Trilogy® Laboratory Fluorometer with CDOM/NH_4 module, Turner Designs Inc.) and following >4 h incubation with OPA in the dark (LOD = 0.094 µM NH_4^+).

For the calculation of fluxes within the benthic incubations chambers, discrete water samples for dissolved inorganic carbon (DIC), total alkalinity (TA), DOC, and DIN were withdrawn from the sampling ports with acid-washed syringes at the beginning and the end of each incubation. Subsamples for DIN were processed and measured according to the protocols outlined above and in Roth et al. (2018). Directly after sampling, subsamples for DIC and TA were transferred into 100 mL borosilicate bottles and immediately poisoned with saturated mercury chloride ($HgCl_2$) to inhibit biological activity. DIC was measured using an inorganic carbon analyzer (Apollo SciTech, AS-C3) equipped with a non-dispersive infrared gas analyzer (LI-COR, Li-7000 CO_2/H_2O). TA was measured by open-cell potentiometric acid-titration (Mettler Toledo, T50). Procedures for DIC and TA measurements were performed according to Dickson et al. (2007). Precision for both instruments was typically within ± 4 µmol kg^{-1}.Subsamples for DOC were filtered through 0.2 µm Millipore® polycarbonate filters into pre-combusted (450°C, 4.5 h) acid-washed amber glass vials (Wheaton) with Teflon-lined lids, and samples were subsequently acidified with H_3PO_4 until reaching a pH 1 – 2. Samples were kept in the dark at 4°C until further analysis by high-temperature catalytic oxidation (HTCO) using a total organic carbon

analyzer (Shimadzu, TOC-L). Reference material of low carbon water (1 – 2 mmol C L^{-1}) and deep-sea carbon (42 – 45 mmol C L^{-1}) were used to monitor the accuracy of concentration measurements. Before sampling, all collection and sample processing devices for DOC (except the polycarbonate filters) were submerged in a 4% HCl bath to leach out any potential contamination of DOC samples.

Section S2: Assessment of benthic community composition

The community composition of each community used for *in situ* incubations was assessed three times during the study period (i.e., at the beginning, after six months, and at the end). The three-dimensional (3D) surface area and the volume of each community was quantified by photogrammetric techniques involving computer modelling (Lavy et al. 2015, Gutierrez-Heredia et al. 2016, Roth et al. 2019). The 3D images of the communities were used to derive the percent cover of the major functional groups (i.e., hard coral, soft coral, turf algae, biogenic rock, rubble, sediment, and macroalgae), encompassing 98% of the visible substrate of the communities. The rest (e.g., other sessile invertebrates or organisms that could not be identified) were grouped into "other". Principal coordinates analysis (PCoA) was used to explore and to visualize dissimilarities of the community composition data using the Bray Curtis dissimilarity (Figure S1). Two-factor permutational multivariate analysis of variance (PERMANOVA) was used to test for the significance of the differences observed between treatments (i.e., coral- vs algae-dominated communities) and to analyze the change in community structure over time (i.e., in the beginning, after six months, and at the end of the experiments). Multivariate dispersion between groups of samples (factor levels) was tested using the function betadisper from the "vegan" R package. The multivariate dispersion was similar between groups of samples regarding "treatment" and "sampling" (Table S1). PERMANOVA results indicated that differences between communities grouped according to coral and algal dominance were significant ($P = 0.001$), while no significant changes over time were detected (Table S1).

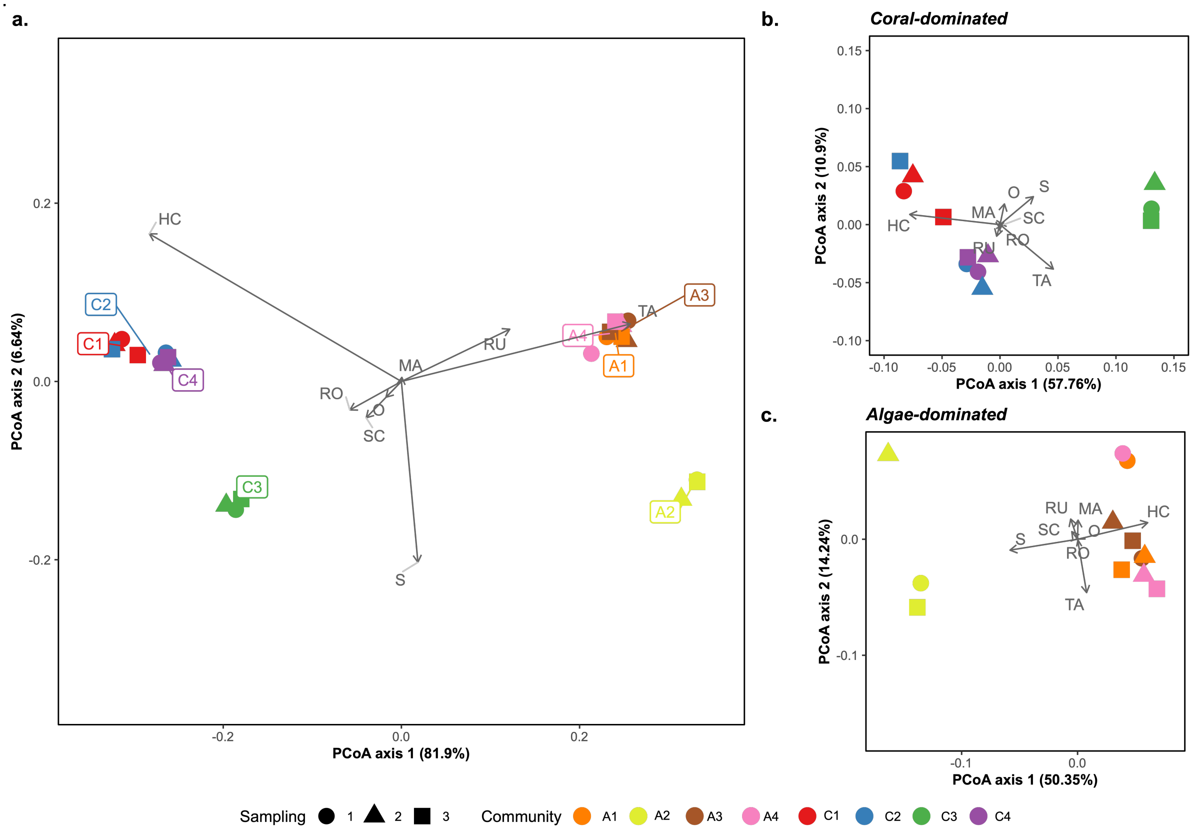

Figure S1: Principal Coordinates Analysis (PCoA) of benthic communities used for *in situ* incubations. PCoA for coral- and algae-dominated communities together (a), and coral- (b), and algae-dominated (c) communities individually. The PCoA plots were based on Bray-Curtis distance and the relative benthic cover of functional groups. Points that are closer together on the ordination have more similar communities. Functional groups as vectors associated to the PCoA: HC = hard coral; SC = soft coral; TA = turf algae; RO = biogenic rock; RU = rubble; S = sediment; MA = macroalgae; O = other. Different colors of symbols represent one community each, where A1 – A4 relate to algae-dominated communities, and C1 – C4 relate to coral-dominated communities. Each community was assessed three times during the study period; differently shaped symbols depict the different time-points. Close-ups to the right refer to inserts in the main plot.

Table S1. Two-factor permutational analysis of variance (PERMANOVA) and dispersion (PERMDISP) results for the community compositions. The factors were ‘Treatment’ (i.e., coral- vs algae-dominated communities) and ‘Sampling’ (i.e., the assessment in the beginning, after six months, and at the end of the study period).

	PERMANOVA					PERMDISP		
	Df	SS	R2	F	Pr(>F)	Df	F	Pr(>F)
Treatment	1	1.182	0.917	205.668	0.001	1	0.1867912	0.667
Sampling	2	0.002	0.002	0.196	0.875	2	0.0322046	0.972
Treatment x Sampling	2	0.001	0.001	0.076	0.954			
Residual	18	0.103	0.08	NA	NA			

Table S2. Monthly measurements of environmental variables in the research area at 5 m water depth. Temperature was measured continuously (logging interval = 30 min) during the whole study period. Light availability was measured continuously (logging interval = 1 minute) at three consecutive days each month, and values are presented as daytime means. All other parameters were measured monthly at three consecutive days. PAR = Photosynthetic active radiation; DIN = Dissolved inorganic nitrogen (NO_3^- + NO_2 + NH_4^+). Values are means ± SE. Adapted from (Roth et al. 2018).

	**Temperature** [°C]	SE	**Salinity**	SE	**PAR** [µmol photons $m^{-2}s^{-1}$]	SE	**Nitrate** [µM NO_3^-]	SE	**Nitrite** [µM NO_2]	SE	**Ammonia** [µM NH_4^+]	SE	**DIN** [µM N]	SE	**Phosphate** [µM PO_4^{3-}]	SE	**Silicate** [µM $Si(OH)_4$]	SE
**January 2017**	26.68	0.06	39.7	0.0	126	12	0.25	0.05	0.03	0.00	0.16	0.01	0.44	0.01	0.02	0.01	0.56	0.00
**February**	25.01	0.04	39.6	0.1	213	17	0.36	0.08	0.04	0.01	0.17	0.03	0.58	0.01	0.08	0.01	0.66	0.04
**March**	25.40	0.06	39.6	0.1	261	26	0.40	0.06	0.06	0.01	0.16	0.02	0.61	0.04	0.21	0.00	0.74	0.02
**April**	27.22	0.14	39.7	0.1	348	29	0.18	0.04	0.03	0.01	0.08	0.02	0.29	0.01	0.11	0.02	0.71	0.03
**May**	29.42	0.16	39.7	0.1	384	18	0.28	0.08	0.02	0.00	0.06	0.03	0.36	0.03	0.04	0.00	0.75	0.04
**June**	30.44	0.05	39.7	0.0	397	20	0.41	0.07	0.04	0.01	0.14	0.02	0.59	0.02	0.10	0.02	0.69	0.04
**July**	31.87	0.09	39.6	0.1	551	13	0.45	0.06	0.05	0.02	0.21	0.06	0.71	0.04	0.18	0.02	0.81	0.05
**August**	32.11	0.03	40.0	0.0	595	14	0.42	0.04	0.04	0.02	0.17	0.02	0.63	0.03	0.12	0.02	0.71	0.07
**September**	32.10	0.07	39.9	0.0	366	28	1.07	0.13	0.06	0.01	0.22	0.03	1.35	0.12	0.16	0.04	0.92	0.07
**October**	31.04	0.05	40.2	0.0	309	5	0.94	0.09	0.04	0.02	0.16	0.04	1.10	0.09	0.15	0.03	0.87	0.06
**November**	29.88	0.15	40.1	0.0	140	11	0.93	0.10	0.04	0.01	0.11	0.01	1.08	0.11	0.11	0.01	1.01	0.01
**December**	27.41	0.04	39.9	0.0	76	7	0.78	0.09	0.05	0.01	0.10	0.02	0.89	0.13	0.12	0.02	0.86	0.03
**January 2018**	25.73	0.07	39.9	0.1	131	6	0.74	0.02	0.05	0.03	0.10	0.03	0.80	0.10	0.12	0.03	0.80	0.02

Table S3: Measured* (TA, DIC) and calculated (pH$_T$, pCO$_2$, Ω$_{arag}$) parameters of the carbonate system for the months of sampling. Values presented as averages ± SE (n = 6) of discrete water samples taken as controls in the research area at 5 m water depth, before the start (around 9:00 a.m.) of experimental *in situ* incubations. Calculated system parameters were derived with measured salinity, temperature, nutrients (phosphate and silicate), total alkalinity (TA), and dissolved inorganic carbon (DIC) data using the CO2SYS Microsoft Excel Macro by Pierrot, Lewis, & Wallace (2006). Dissociation constants for carbonate (K1 and K2) were chosen from Mehrbach, Culberson, Hawley, & Pytkowicx (1973), determined as refit by Dickson & Millero (1987) and the dissociation constant for boric acid determined by Dickson (1990). pH is reported on the total scale, referred to as pH$_T$, the scale on which K1 and K2 were determined in the Gran functions.

	**TA*** [µmol kg^{-1}]	SE	**DIC*** [µmol kg^{-1}]	SE	**pH$_T$**	SE	***p*CO$_2$** [µatm]	SE	**Ω$_{arag}$**	SE
**January 2017**	2389	4	2001	3	8.06	0.01	378	7	4.2	0.1
**March**	2383	6	1934	12	8.15	0.02	296	17	4.8	0.1
**May**	2364	6	1859	4	8.16	0.00	265	5	5.5	0.3
**July**	2411	3	1916	2	8.12	0.01	304	4	5.5	0.0
**September**	2379	3	1907	3	8.10	0.00	328	3	5.2	0.0
**November**	2375	6	1940	1	8.10	0.01	341	7	4.7	0.1
**January 2018**	2385	7	1988	7	8.11	0.02	342	17	4.2	0.1

Table S4. Results from linear mixed models (LMMs) and Tukey HSD all pairwise comparisons of response parameters. Response parameters from incubations (net community production - NCP, community respiration - CR, gross primary production - GPP, GPP/CR, net community calcification - NCC, dissolved organic carbon fluxes - DOC, and dissolved inorganic nitrogen fluxes – DIN) were assessed by LMMs, with 'treatment' (coral- vs. algae-dominated) and 'season' (spring, summer, fall, and winter) as fixed factors. The sampling dates (date) within seasons and the replicates (community ID) were random factors. If significant interactions (treatment*season) were found, Tukey HSD all pairwise comparisons were used to determine which means differed. Abbreviations: Std error = standard error for the covariance component estimate; DFDen = denominator degrees of freedom; t ratio = ratio of the estimate to its standard error; Prob>|t| = p-value for a two-sided test of the t ratio; 95% Lower = lower 95% confidence limit; 95% Upper = upper 95% confidence limit; Nparm = number of parameters associated with the effec; DFNum = numerator degrees of freedom for the effect test.

NCP [mmol C m^{-2} d^{-1}]

Fixed Effects Parameter Estimates

Term	Estimate	Std error	DFDen	t ratio	Prob>|t|	95% Lower	95% Upper
Intercept	381.51094	4.2110122	0.2	90.6	0.2531	-375327.6	376090.58
Season[Fall]	-0.423438	15.602825	3	-0.03	0.9801	-50.07876	49.231881
Season[Spring]	14.676563	12.276237	3	1.2	0.3178	-24.39203	53.745158
Season[Summer]	10.395313	12.276237	3	0.85	0.4593	-28.67328	49.463908
Treatment[Algae]	64.935937	5.6054504	9.2	11.58	<.0001*	52.294575	77.5773
Season[Fall]*Treatment[Algae]	23.926562	17.347612	39	1.38	0.1757	-11.16229	59.01542
Season[Spring]*Treatment[Algae]	-22.18594	13.649028	39	-1.63	0.1121	-49.7937	5.4218278
Season[Summer]*Treatment[Algae]	95.895313	13.649028	39	7.03	<.0001*	68.287547	123.50308

Fixed Effects Tests

Source	Nparm	DFNum	DFDen	F ratio	Prob > F
Season	3	3	3	1.6732526	0.3414
Treatment	1	1	9.2	134.19898	<.0001*
Season*Treatment	3	3	39	28.299645	<.0001*

Tukey HSD All Pairwise Comparisons

Season	Treatment	-Season	-Treatment	Difference	Std error	t ratio	Prob>|t|	Lower 95%	Upper 95%
Fall	Algae	Fall	Coral	177.725	40.90689	4.34	0.0022*	46.787	308.663
Fall	Algae	Winter	Algae	145.787	35.27485	4.13	0.0041*	32.877	258.698
Fall	Coral	Summer	Algae	-260.513	32.91489	-7.91	<.0001*	-365.869	-155.156
Spring	Algae	Summer	Algae	-113.800	28.80179	-3.95	0.0070*	-205.991	-21.609
Spring	Algae	Winter	Algae	114.775	28.80179	3.98	0.0063*	22.584	206.966
Spring	Coral	Summer	Coral	122.363	28.80179	4.25	0.0030*	30.172	214.553
Summer	Algae	Summer	Coral	321.663	27.49954	11.7	<.0001*	233.64	409.685
Summer	Algae	Winter	Algae	228.575	28.80179	7.94	<.0001*	136.384	320.766
Summer	Coral	Winter	Coral	-158.488	28.80179	-5.50	<.0001*	-250.678	-66.297

GPP [mmol C m^{-2} d^{-1}]

Fixed Effects Parameter Estimates

Term	Estimate	Std error	DFDen	t ratio	Prob>|t|	95% Lower	95% Upper
Intercept	761.71719	23.925011	2.9	31.84	<.0001*	684.04924	839.38514
Season[Fall]	-11.80469	49.03662	3	-0.24	0.8253	-167.8611	144.25172
Season[Spring]	44.289063	38.581806	3	1.15	0.3342	-78.49546	167.07359
Season[Summer]	137.68281	38.581806	3	3.57	0.0376*	14.898286	260.46734
Treatment[Algae]	197.52969	12.377472	7.2	15.96	<.0001*	168.40095	226.65843
Season[Fall]*Treatment[Algae]	105.80781	25.375811	39	4.17	0.0002*	54.48039	157.13523
Season[Spring]*Treatment[Algae]	-29.56094	19.965581	39	-1.48	0.1467	-69.94514	10.823262
Season[Summer]*Treatment[Algae]	126.58281	19.965581	39	6.34	<.0001*	86.198613	166.96701

Fixed Effects Tests

Source	Nparm	DFNum	DFDen	F ratio	Prob > F
Season	3	3	3	9.0852985	0.0514
Treatment	1	1	7.2	254.68353	<.0001*
Season*Treatment	3	3	39	42.691291	<.0001*

Tukey HSD All Pairwise Comparisons

Season	Treatment	-Season	-Treatment	Difference	Std error	t ratio	Prob>|t|	Lower 95%	Upper 95%
Fall	Algae	Fall	Coral	606.675	62.64548	9.68	<.0001*	406.15	807.195
Fall	Algae	Winter	Algae	467	83.47485	5.59	<.0001*	199.81	734.192
Spring	Algae	Spring	Coral	335.938	44.29444	7.58	<.0001*	194.16	477.718
Spring	Algae	Winter	Algae	387.725	68.15693	5.69	<.0001*	169.56	605.887
Summer	Algae	Summer	Coral	648.225	44.29444	14.63	<.0001*	506.44	790.006
Summer	Algae	Winter	Algae	637.263	68.15693	9.35	<.0001*	419.1	855.424

CR [mmol C m^{-2} d^{-1}]

Fixed Effects Parameter Estimates

Term	Estimate	Std error	DFDen	t ratio	Prob>|t|	95% Lower	95% Upper
Intercept	-380.2093	18.738732	3	-20.29	0.0003*	-439.8129	-320.6058
Season[Fall]	11.396845	38.280154	3	0.3	0.7853	-110.4277	133.22138
Season[Spring]	-29.61565	30.118664	3	-0.98	0.398	-125.4667	66.235377
Season[Summer]	-127.2907	30.118664	3	-4.23	0.0242*	-223.1417	-31.43962
Treatment[Algae]	-132.6026	7.418273	7.1	-17.88	<.0001*	-150.087	-115.1182
Season[Fall]*Treatment[Algae]	-81.90987	14.889966	39	-5.50	<.0001*	-112.0277	-51.79207
Season[Spring]*Treatment[Algae]	7.3776262	11.715363	39	0.63	0.5325	-16.31893	31.074184
Season[Summer]*Treatment[Algae]	-30.67237	11.715363	39	-2.62	0.0125*	-54.36893	-6.975816

Fixed Effects Tests

Source	Nparm	DFNum	DFDen	F ratio	Prob > F
Season	3	3	3	11.437412	0.0378*
Treatment	1	1	7.1	319.52042	<.0001*
Season*Treatment	3	3	39	29.478208	<.0001*

Tukey HSD All Pairwise Comparisons

Season	Treatment	-Season	-Treatment	Difference	Std error	t ratio	Prob>|t|	Lower 95%	Upper 95%
Fall	Algae	Fall	Coral	-429.025	36.88294	-11.63	<.0001*	-547.083	-310.967
Fall	Algae	Winter	Algae	-321.227	62.0982	-5.17	0.0002*	-519.995	-122.459
Spring	Algae	Spring	Coral	-250.450	26.16602	-9.57	<.0001*	-334.204	-166.696
Spring	Algae	Winter	Algae	-272.952	50.70296	-5.38	<.0001*	-435.246	-110.658
Summer	Algae	Summer	Coral	-326.550	26.16602	-12.48	<.0001*	-410.304	-242.796
Summer	Algae	Winter	Algae	-408.677	50.70296	-8.06	<.0001*	-570.971	-246.383

GPP/CR

Fixed Effects Parameter Estimates

Term	Estimate	Std error	DFDen	t ratio	Prob>|t|	95% Lower	95% Upper
Intercept	2.1984375	0.0582625	2.2	37.73	0.0004*	1.9673102	2.4295648
Season[Fall]	0.1640625	0.1284201	3	1.28	0.2913	-0.244628	0.5727532
Season[Spring]	-0.110937	0.1010404	3	-1.10	0.3524	-0.432494	0.2106186
Season[Summer]	-0.467188	0.1010404	3	-4.62	0.0190*	-0.788744	-0.145631
Treatment[Algae]	-0.264063	0.02895	8.1	-9.12	<.0001*	-0.330704	-0.197421
Season[Fall]*Treatment[Algae]	-0.298438	0.0758602	39	-3.93	0.0003*	-0.451879	-0.144996
Season[Spring]*Treatment[Algae]	0.0140625	0.0596865	39	0.24	0.815	-0.106665	0.1347898
Season[Summer]*Treatment[Algae]	0.3453125	0.0596865	39	5.79	<.0001*	0.2245852	0.4660398

Fixed Effects Tests

Source	Nparm	DFNum	DFDen	F ratio	Prob > F
Season	3	3	3	10.97097	0.0399*
Treatment	1	1	8.1	83.198429	<.0001*
Season*Treatment	3	3	39	12.264361	<.0001*

Tukey HSD All Pairwise Comparisons

Season	Treatment	-Season	-Treatment	Difference	Std error	t ratio	Prob>|t|	Lower 95%	Upper 95%
Fall	Algae	Fall	Coral	-1.12500	0.1815169	-6.20	<.0001*	-1.70601	-0.54399
Fall	Coral	Summer	Coral	1.275	0.2254975	5.65	<.0001*	0.55321	1.99679
Spring	Algae	Spring	Coral	-0.50000	0.1241372	-4.03	0.0056*	-0.89735	-0.10265
Spring	Coral	Summer	Coral	0.6875	0.184118	3.73	0.0127*	0.09816	1.27684
Spring	Coral	Winter	Coral	-0.60000	0.184118	-3.26	0.0434*	-1.18934	-0.01066
Summer	Coral	Winter	Coral	-1.28750	0.184118	-6.99	<.0001*	-1.87684	-0.69816

NCC [mmol $CaCO_3$ m^{-2} d^{-1}]

Fixed Effects Parameter Estimates

Term	Estimate	Std error	DFDen	t ratio	Prob>|t|	95% Lower	95% Upper
Intercept	73.7875	2.4698256	1.1	29.88	0.0155*	48.421986	99.153014
Season[Fall]	21.8	5.7167961	3	3.81	0.0317*	3.6068217	39.993178
Season[Spring]	11.00625	4.4979511	3	2.45	0.0919	-3.308066	25.320566
Season[Summer]	-36.75625	4.4979511	3	-8.17	0.0038*	-51.07057	-22.44193
Treatment[Algae]	-62.9375	3.0167456	7.4	-20.86	<.0001*	-69.99022	-55.88478
Season[Fall]*Treatment[Algae]	-17.425	6.7293953	39	-2.59	0.0134*	-31.03649	-3.813513
Season[Spring]*Treatment[Algae]	-14.48125	5.2946599	39	-2.74	0.0093*	-25.19071	-3.771789
Season[Summer]*Treatment[Algae]	38.49375	5.2946599	39	7.27	<.0001*	27.784289	49.203211

Fixed Effects Tests

Source	Nparm	DFNum	DFDen	F ratio	Prob > F
Season	3	3	3	22.642704	0.0146*
Treatment	1	1	7.4	435.25284	<.0001*
Season*Treatment	3	3	39	18.017907	<.0001*

Tukey HSD All Pairwise Comparisons

Season	Treatment	-Season	-Treatment	Difference	Std error	t ratio	Prob>|t|	Lower 95%	Upper 95%
Fall	Algae	Fall	Coral	-160.725	16.41024	-9.79	<.0001*	-213.252	-108.198
Fall	Coral	Summer	Coral	114.475	13.3495	8.58	<.0001*	71.745	157.205
Spring	Algae	Spring	Coral	-154.838	11.45798	-13.51	<.0001*	-191.513	-118.162
Spring	Coral	Summer	Coral	100.738	10.89982	9.24	<.0001*	65.849	135.626
Summer	Algae	Summer	Coral	-48.888	11.45798	-4.27	0.0028*	-85.563	-12.212
Summer	Coral	Winter	Coral	-85.787	10.89982	-7.87	<.0001*	-120.676	-50.899

DOC [mmol C m^{-2} d^{-1}]

Fixed Effects Parameter Estimates

Term	Estimate	Std error	DFDen	t ratio	Prob>|t|	95% Lower	95% Upper
Intercept	9.1572736	1.8213858	3.2	5.03	0.0126*	3.5970814	14.717466
Season[Fall]	0.086428	3.609331	3	0.02	0.9824	-11.40007	11.57293
Season[Spring]	-1.302485	2.8398064	3	-0.46	0.6777	-10.34002	7.7350469
Season[Summer]	3.8019156	2.8398064	3	1.34	0.273	-5.235616	12.839447
Treatment[Algae]	-5.418692	1.0451287	6.9	-5.18	0.0013*	-7.896322	-2.941062
Season[Fall]*Treatment[Algae]	-1.572893	1.9187576	39	-0.82	0.4173	-5.453947	2.3081606
Season[Spring]*Treatment[Algae]	-0.872545	1.5096704	39	-0.58	0.5666	-3.926142	2.1810515
Season[Summer]*Treatment[Algae]	-0.480754	1.5096704	39	-0.32	0.7518	-3.53435	2.5728428

Fixed Effects Tests

Source	Nparm	DFNum	DFDen	F ratio	Prob > F
Season	3	3	3	0.7670749	0.5837
Treatment	1	1	6.9	26.881245	0.0013*
Season*Treatment	3	3	39	1.261455	0.3009

Random Effects Covariance Parameter Estimates

Variance Component	Estimate	Std error	95% Lower	95% Upper
Date	14.240974	16.258051	-17.62422	46.106169
Community ID	1.7257218	4.9172976	-7.912004	11.363448

DIN [mmol N m^{-2} d^{-1}]

Fixed Effects Parameter Estimates

Term	Estimate	Std error	DFDen	t ratio	Prob>|t|	95% Lower	95% Upper
Intercept	1.7224734	0.259769	5.7	6.63	0.0007*	1.0790532	2.3658936
Season[Fall]	0.5533765	0.3824594	3	1.45	0.2437	-0.66378	1.7705331
Season[Spring]	-0.727328	0.3009175	3	-2.42	0.0944	-1.684981	0.230326
Season[Summer]	0.3478928	0.3009175	3	1.16	0.3313	-0.609761	1.3055465
Treatment[Algae]	0.0122919	0.2227188	6.4	0.06	0.9577	-0.525163	0.5497467
Season[Fall]*Treatment[Algae]	-0.031205	0.2668239	39	-0.12	0.9075	-0.570907	0.5084973
Season[Spring]*Treatment[Algae]	0.278539	0.2099359	39	1.33	0.1923	-0.146096	0.7031744
Season[Summer]*Treatment[Algae]	-0.186161	0.2099359	39	-0.89	0.3806	-0.610796	0.2384746

Fixed Effects Tests

Source	Nparm	DFNum	DFDen	F ratio	Prob > F
Season	3	3	3	2.427236	0.2428
Treatment	1	1	6.4	0.003046	0.9577
Season*Treatment	3	3	39	0.715285	0.5488

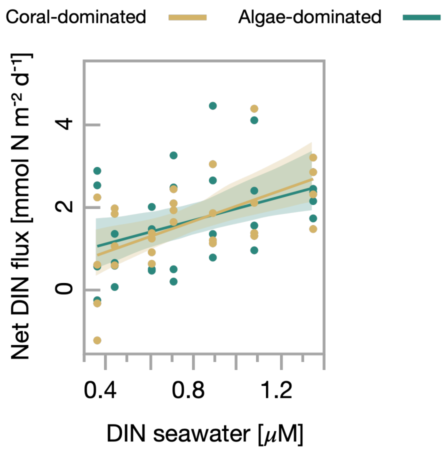

Figure S2: Relationship between net dissolved inorganic nitrogen (DIN) fluxes and DIN concentrations in seawater in coral and algae-dominated reef communities. Shaded areas in transparent colors represent the 95% confidence intervals around the lines of the estimates.

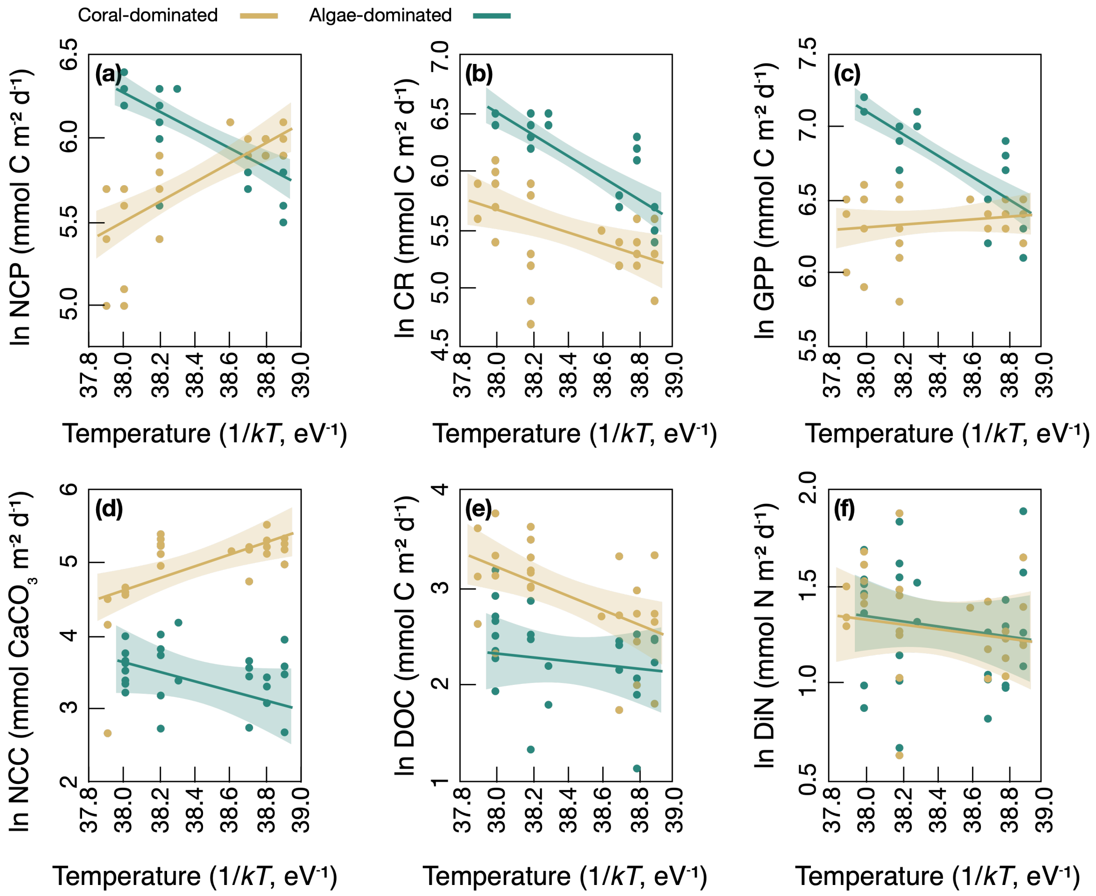

Figure S3: Arrhenius plots showing the relationship between the inverted temperature multiplied by the Boltzmann constant (1/kT) and the natural logarithm of (a) net community production (NCP), (b) community respiration (CR), (c) gross primary production (GPP), (d) net community calcification (NCC), (e) dissolved organic carbon (DOC) fluxes, and (f) dissolved inorganic nitrogen (DIN) fluxes for coral- and algae-dominated reef communities. Solid lines indicate the linear regression, shaded areas in transparent colors represent the 95% confidence intervals.

Table S5: Compilation of *in situ* measurements of community metabolism from different coral reef environments around the world. NCP = Net community production; NCC = Net community calcification. Note that not all studies measured all parameters.

Community type and location	Study	Approach	NCP			NCC		
			Light [mmol C m-2 h-1]	Dark [mmol C m-2 h-1]	24 h NET flux [mmol C m-2 d-1]	Light [mmol CaCO3 m-2 h-1]	Dark [mmol CaCO3 m-2 h-1]	24 h NET flux [mmol CaCO3 m-2 d-1]
Coral-dominated community Central Red Sea, Saudi Arabia	This study	In situ incubation	**26.68** ±1.24	**-10.87** ±0.71	**189.58** **±19.02**	**7.93** ±0.73	**3.01** ±0.34	**131.12** ±10.1
Algae-dominated community Central Red Sea, Saudi Arabia	This study	In situ incubation	**36.92** ±1.72	**-20.94** ±1.36	**191.72** **±11.79**	**1.32** ±0.21	**-0.47** ±0.18	**10.23** ±2.63
Algae-dominated coral reef Central Red Sea, Saudi Arabia	(Bernstein et al. 2016)	Eulerian flow respirometry	21 to 44			8 to 11		
Reef flat Heron Island, Australia	(Albright et al. 2015)	Lagrangian approach	11 to 61	-28 to -3	-6	3 to 15	-2 to 5	104
Reef flats global overview	Atkinson (2011)				-220 to 310			20 to 250
Lagoon	(McMahon et al. 2013)	Lagrangian approach	38.4	-27.4	132	8.5	-3.7	57.6
Algae-dominated reef flat Davies Reef, Australia	(Albright et al. 2013)	Lagrangian approach	9 to 64	-7 to -30		5 to 17	-3 to 7	77 to 166

References:

Albright, R., J. Benthuysen, N. Cantin, K. Caldeira, and K. Anthony. 2015. Coral reef metabolism and carbon chemistry dynamics of a coral reef flat. Geophysical Research Letters 42:3980–3988.

Albright, R., C. Langdon, and K. R. N. Anthony. 2013. Dynamics of seawater carbonate chemistry, production, and calcification of a coral reef flat, Central Great Barrier Reef. Biogeosciences Discussions 10:7641–7676.

Bernstein, W. N., K. A. Hughen, C. Langdon, D. C. McCorkle, and S. J. Lentz. 2016. Environmental controls on daytime net community calcification on a Red Sea reef flat. Coral Reefs 35:697–711.

Dickson, A. G. 1990. Thermodynamics of the dissociation of boric acid in synthetic seawater from 273.15 to 318.15 K. Deep Sea Research Part A. Oceanographic Research Papers 37:755–766.

Dickson, A. G., and F. J. Millero. 1987. A comparison of the equilibrium constants for the dissociation of carbonic acid in seawater media. Deep Sea Research Part A. Oceanographic Research Papers 34:1733–1743.

Dickson, A. G., C. L. Sabine, and J. R. Christian. 2007. Guide to Best Practices for Ocean CO2 Measurments. North Pacific Marine Science Organization.

Grasshoff, K., M. Ehrhardt, K. Kremling, and L. G. Anderson. 1999. Methods of seawater analysis. Wiley-VCH.

Gutierrez-Heredia, L., F. Benzoni, E. Murphy, and E. G. Reynaud. 2016. End to End Digitisation and Analysis of Three-Dimensional Coral Models, from Communities to Corallites. Plos One 11:e0149641.

Holmes, R. M., A. Aminot, R. Kerouel, B. A. Hooker, and B. J. Peterson. 1999. A simple and precise method for measuring ammonium in marine and freshwater ecosystems. Canadian Journal of Fisheries and Aquatic Sciences 56:1801–1808.

Lavy, A., G. Eyal, B. Neal, R. Keren, Y. Loya, and M. Ilan. 2015. A quick, easy and non-intrusive method for underwater volume and surface area evaluation of benthic organisms by 3D computer modelling. Methods in Ecology and Evolution 6:521–531.

McMahon, A., I. R. Santos, T. Cyronak, and B. D. Eyre. 2013. Hysteresis between coral reef calcification and the seawater aragonite saturation state. Geophysical Research Letters 40:4675–4679.

Mehrbach, C., C. H. Culberson, J. E. Hawley, and R. M. Pytkowicx. 1973. MEASUREMENT OF THE APPARENT DISSOCIATION CONSTANTS OF CARBONIC ACID IN SEAWATER AT ATMOSPHERIC PRESSURE1. Limnology and Oceanography 18:897–907.

Pierrot, D., E. Lewis, and D. W. R. Wallace. 2006. MS Excel Program Developed for CO2 System Calculations. Oak Ridge.

Roth, F., F. Saalmann, T. Thomson, D. J. Coker, R. Villalobos, B. H. Jones, C. Wild, and S. Carvalho. 2018. Coral reef degradation affects the potential for reef recovery after disturbance. Marine Environmental Research 142:48–58.

Roth, F., C. Wild, S. Carvalho, N. Rädecker, C. R. Voolstra, B. Kürten, H. Anlauf, Y. C. El-Khaled, R. Carolan, and B. H. Jones. 2019. An in situ approach for measuring biogeochemical fluxes in structurally complex benthic communities. Methods in Ecology and Evolution 10:712–725.

Taylor, B. W., C. F. Keep, R. O. Hall, B. J. Koch, L. M. Tronstad, A. S. Flecker, and A. J. Ulseth. 2007. Improving the fluorometric ammonium method: matrix effects, background fluorescence, and standard additions. Journal of the North American Benthological Society 26:167–177.
